# Natural hazard mitigation strategies review: Actor–network theory and the eco-based approach understanding in Zimbabwe

**DOI:** 10.4102/jamba.v11i1.629

**Published:** 2019-01-16

**Authors:** Anyway Katanha, Danny Simatele

**Affiliations:** 1 Department of Geography and Environmental Studies, University of the Witwatersrand, South Africa

## Abstract

This paper presents the literature reviewed on the evolution of the natural hazard mitigation perspective and an overview of its progression to date. The article uses information taken from diverse sources such as a globally accepted scientific databases Google Scholar (http://www.scholar.google.co.in), Scopus (http://www.scopus.com), Science Direct (http://www.sciencedirect.com), SpringerLink (http://www.springer.co.in) and Wiley (http://www.onlinelibrary.wiley.com); conference proceedings; theses; abstracts; and impact and non-indexed journals. It demonstrates how the actor–network theory (ANT) theoretical framework can be applicable to Muzarabani in Zimbabwe as a tool for analysing and elaborating hazard mitigation strategies. Actor–network theory is gradually becoming influential but is still a bone of contention, mainly because of its radical approach. Actor–network theory treats humans and non-humans as equal actors. In spite of its limitations, studies have shown that an ANT-grounded approach is useful in providing a framework for the comprehension of the complexities of daily life during natural hazard episodes and the dynamic role of *Ziziphus mauritiana* in the network in Muzarabani, Zimbabwe. The theory can demonstrate its importance in respect of how social results are produced as a result of linkages among diverse actors (human and non-human) in a network. The article argues that if ANT is used logically it is useful in examining eco-based natural hazard mitigation and resilience approaches in semi-arid regions.

## Introduction

The purpose of this paper is to critically review theoretical hazard mitigation evolution, concepts and approaches. Comprehensive hazard documentation is vital for the examination of precedent events, their impacts and responses (Kreibich et al. 2005; Schröter et al. 2015). The review is contextualised to fit a global perspective, highlighting the African experience in semi-arid zones (Quandt et al. 2017). For instance, developing countries experience a higher loss of life, while developed countries experience more economic losses (Mileti 1999). However, similar concerns exist around questions about the impacts and experiences. When questions like ‘who is impacted?’ are asked, how does one respond to the difficult experiences caused by hazards using available natural resources and other actors? In the course of this review, an overview of structural and non-structural hazard mitigation approaches is discussed (Mileti 1999). This is followed by a summary of the role of *Ziziphus mauritiana* and the potential of actor–network theory (ANT) to enhance the understanding of hazard mitigation options. The review leads to an argument of recent unique understandings of the role of both human and non-human actors in any process. The ANT is explored from an ecological service perspective, arguing that in order for hazard mitigation and resilience to be effective, it must be considered together with other actors in the discourse.

## Methodological consideration

**FIGURE 1:**
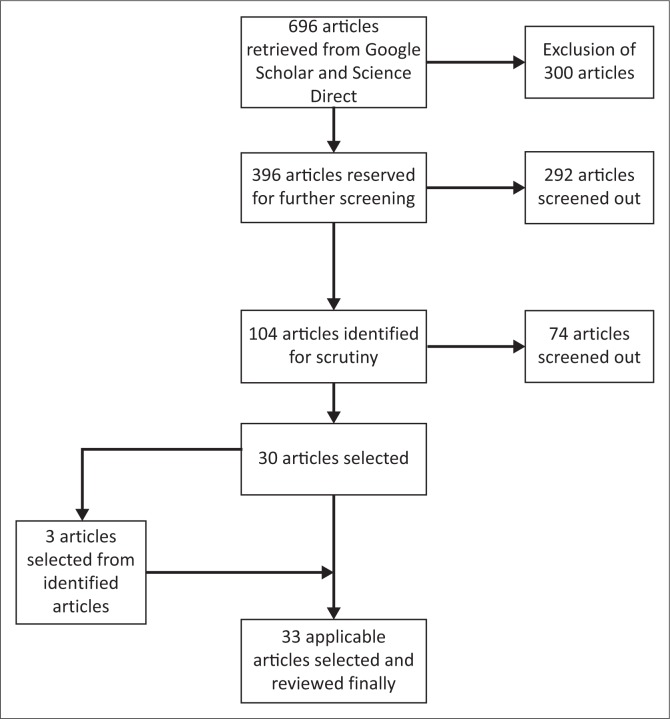
Selection and screening of publication process flow chart.

A systematic search to retrieve related scholarly literature and referenced articles was conducted between January 2015 and September 2016. The main aim of the research was to identify natural hazard mitigation approaches and ecological studies in semi-arid regions. The search paid particular attention to *Ziziphus mauritiana* and its interaction with different human and non-human actors in semi-arid regions. The inclusion and exclusion criteria were discussed and agreed on at the commencement of the selection process (Creswell 2007). The selection criteria were developed until the final selection conditions were established (Denzin & Lincolan 2000). This enabled the study to eliminate scholarly work that was outside the eco-based natural hazard mitigation approach scope and confirmed consistency. This was achieved through a search on Google Scholar and Science Direct, which helped the study to achieve the elimination process. The keywords were identified and selected from natural hazard and socio-environmental studies and thematic headings. Basic words and terms such as ‘natural hazard mitigation approaches’, ‘climatic variability’, ‘actor–network theory’, ‘eco-resources’, ‘*Ziziphus mauritiana*’, ‘commodity value chain’, ‘Zimbabwe’, ‘Central Africa’, ‘Southern Africa’, ‘developing countries’ and ‘sub-Saharan Africa’ were searched in the engine. The reference list of applicable articles was evaluated to identify other appropriate articles. Figure 1 illustrates the chronological sequence of the search strategy, screening and selection processes. Finally, selected articles were included.

### Approaches to hazard mitigation

The earliest social scientific insight into hazard mitigation comes from a study by Rousseau, who explains that the shock of the 1755 Lisbon earthquake would have been moderate if the city had been sparsely inhabited and if people had evacuated promptly in reaction to the early tremors (Dynes 2000). The ideas of hazard mitigation have evolved and have been developed over the past half-century. Hazard mitigation studies were championed by renowned academic Gilbert Fowler White in 1945, who studied floodplain management as a way of reducing flood loss rather than dependence on structural flood mitigation (Kenney et al. 2015). White’s work is seminal in the critical study of hazard mitigation methods (Mileti 1999). White’s research led to noteworthy hazard policy changes in places such as Bangladesh, Japan, Sri Lanka, Sweden and the United States (Adger et al. 2009; Neisser 2014). In the search for suitable approaches to hazards, recent studies are promoting a shift in emphasis from rescue to proactive methods in order to mitigate the effects of natural hazards (Manyanye 2015; Neisser 2014). Hazard mitigation strategies have been defined in an array of ways. The most commonly used are the structural and non-structural hazard mitigation strategies (United Nations International Strategy for Disaster Reduction [UNISDR] 2007).

### Structural approaches

Structural mitigation involves making use of engineered protection to afford protection from hazard impacts (UNISDR 2007). Common examples of structural mitigation include road, bridge, dam, dyke and building designs that incorporate construction methods that enhance the ability of structures to withstand hazardous forces. The use of structural approaches to hazard mitigation to some extent saves lives (Mileti 1999). This approach can enable movement of nature instead of people movement. However, the structural hazard mitigation approach involves modification of the physical and natural environment, causing it physical harm and ruin (Enete & Amusa 2010). In the same vein, more losses can be incurred, as structural hazard mitigation approaches present a false sense of security to civilians (Tompkins & Adger 2004). Traditionally, disaster response primarily focused on structural mitigation measures, despite the repeated warnings from scholars that such measures alone were inadequate.

### Non-structural approaches

Non-structural hazard mitigation approaches refer to measures that pursue or help to reduce the possibility or consequence of risk through modifying human action, or natural processes. The following are examples of non-structural hazard mitigation approaches: regulatory or legal measures, awareness and education programmes, natural resource conservation and preservation, and behavioural modification (Tompkins & Adger 2004). Furthermore, non-structural approaches are also comparatively less expensive and provide supplementary sustainable tools to hazard mitigation. However, there is a need for more comprehensive methods that would at least enhance mitigation approaches to hazards (Lindell, Prate & Perry 2006). Several studies on hazard mitigation using non-structural strategies in Bangladesh, Thailand and other countries have been consulted (Burby et al. 1999). Jumping ahead of the scope of what both structural and non-structural approaches can offer are questions concerning how clear hazards interconnect with human and non-human actors, as well as what implications this might have in a hazard mitigation scenario. Against this background there is a paucity of studies that directly seek to improve hazard mitigation approaches, particularly in poor rural communities.

#### Open system approach

Hazard theorisation has been affected by the fact that the discourse is complex in terms of social, ecological and political considerations (Kenney et al. 2015). One theoretical approach that has been used is known as ‘open systems’, introduced by the founder of natural hazards as an interdisciplinary science (Mileti 1999). This approach considers communities and societies as human systems that act in accordance with disturbances. It is also known as the intersection of society and nature theory. This theory is a paradigm shift from the acts of God or fate theory, which blamed God for hazards.

Early uses of the acts of God theory suggested that unfavourable events of a personal nature were the result of an unfavourable configuration of the planets and stars (White 1945). With the passage of time it was applied to major environmental disturbances such as earthquakes, drought and floods (Mileti 1999). The earliest proponents of the acts of God notion believed that a natural hazard was a punishment from God for human sins and failings (White 1945). This notion was applied by many societies in the world, including the Greeks, Romans and Chinese. The only solution to these acts of God was human sacrifice, meaning people could be killed to appease God. Others viewed hazards or disasters as God’s way of naturally maintaining order or regulating human behaviour. Later, acts of God came to be seen as merely the way that things were, or God’s plan, beyond human comprehension (Angenheister 1921).However, this thread of thinking led some to a fatalistic view of a disaster as an act of God, where man could do nothing. Mileti (1999) argued that this approach was not productive because it removed from humans the responsibility of planning to avert human loss and suffering.

#### Political approach

Other approaches have focused on political dynamics. These approaches explain hazard mitigation approaches in terms of their relation to political management styles (Manyanye 2015). The failure of hazard mitigation approaches may be attributed to a blameworthy political management landscape. However, politically based hazard mitigation approaches have been criticised for bias and lack of depth in the analysis of hazard mitigation approaches. Hazard mitigation approaches have, in some instances, focused on information and leadership controls (Mavhura, Manatsa & Mushore 2015; Mileti 1999). Other approaches have focused on intergovernmental structures, because most governments do not have the capacity to implement hazard mitigation options (Mileti 1999; Neisser 2014). Noticeable in all approaches is that mitigation approaches should not be taken for granted; various factors should be considered when implementing measures that are intended to reduce suffering and loss of life in hazard-prone areas. Theoretical and conceptual frameworks need to be developed to reduce the tendency to focus on particular mitigation strategies and to shift from structural to non-structural approaches (White 1973). Hazards, it has been claimed, are acts of God; the loss of life and suffering are a result of mankind’s lack of planning (Mileti 1999).

#### Adaptive capacity or resilience approaches

**FIGURE 2:**
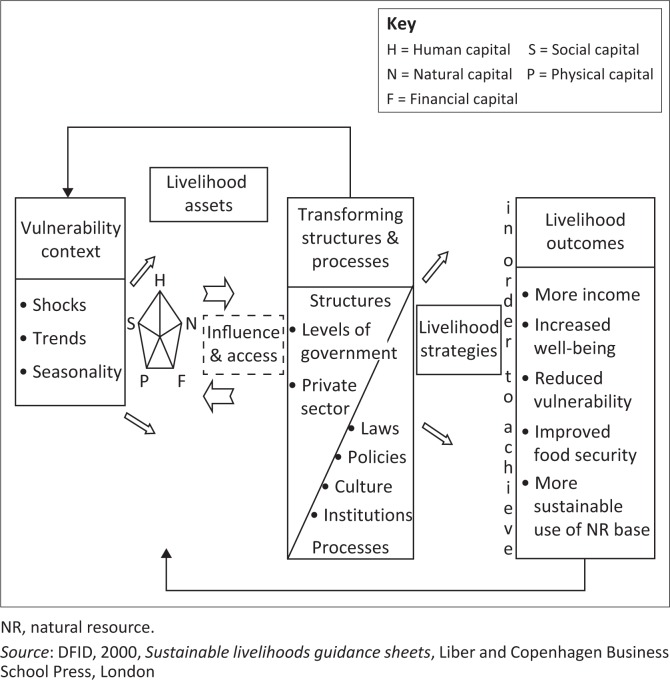
Sustainable livelihoods framework.

According to the Intergovernmental Panel on Climate Change (IPCC) (2013), ‘adaptive capacity’ refers to the adjustment capability of a system to climate variability, to reduce potential harm, to benefit opportunities or to manage the consequences. ‘Adaptive capacity’ is a term commonly used interchangeably with ‘resilience’. A community’s capacity to adapt and survive shocks is based on its resilience (Folke 2006). Resilience varies from one geographical area to another, one community to another. It is determined by the community’s assets and the services provided by the government and other institutions (IPCC 2013; Tompkins & Adger 2004). Assets consist of many things, among which are physical and financial capital, knowledge and labour in a household, social relations and access to natural resources (see Figure 2). Adaptive capacity strategies for societies are shifting from those based on technological developments and engineering structures with economic diversification to strategies based on natural resources (Tompkins & Adger 2004). This confirmation is based on the known association between human livelihoods and ecosystems (Enete & Amusa 2010). Ecosystems provide such benefits as cultural services, food, fibre, fuel and regulating services (Bharucha & Pretty 2010). Indigenous fruit trees like *Z. mauritiana* play an important role in the adaptive capacities of many rural communities affected by extreme weather conditions. Although the comprehensive linkages between indigenous fruits and climate variability are not always well understood, it is widely recognised that they have a role to play in environmental change (Bharucha & Pretty 2010; Quandt et al. 2017).

In most cases the poor rely directly on ecosystem services, especially in areas prone to extreme weather conditions. In Africa, more than 70% of the population depends on rain-fed agriculture, and the rest depends on forest products, fishing and hunting (Quandt et al. 2017). It is against this backdrop that adaptation and mitigation strategies improve resilience and continually provide ecological services and goods that can be vital for poor communities.

#### Sustainable livelihoods framework

The Sustainable Livelihoods Framework of the United Kingdom’s Department for International Development (DFID 2000) presents some of the measures that can be used by communities to adapt to climate variability in Muzarabani. The diagram in Figure 2 shows how communities can operate within a vulnerable environment shaped by different factors and how they draw on different capital, such as natural capital that is interlinked with other capital, like social and economic capital. Figure 2 shows the interrelationship between the different components with the aid of arrows; the ultimate goal is achieving positive livelihoods. In the context of this study, the aim is to enhance the effective roles played by *Z. mauritiana* for the Muzarabani community.

The framework bundles different sectors as complementary adaptation options in rural communities (DFID 2000). The framework is also dynamic as it overlaps in time and space, addressing complex interplay among factors. Although widely adopted by non-governmental organisations (NGOs), the sustainable livelihoods framework has received criticisms (Ncube-Phiri, Mudavanhu & Mucherera 2014). These include its elevated skills and resource requirements. In terms of implementation, little attention is given at times to the complex social ecological consequences of adaptive livelihood. Hence this review introduces a different lens that captures the complexities, namely ANT, explained in the next part of this paper.

## Actor–network theory and hazard mitigation

The literature reviewed points to the fact that few studies have adequately addressed the complex roles of both nature and society in promoting a better understanding of the concept of hazard mitigation (Wessing 1988). Actor–network theory highlights how human and non-human agencies (actors) stimulate the process, guide and edge the insight and action of human users. Actor–network theory concludes with general insights gained, which will be of use in understanding current dynamic social and ecological complexities in alternative hazard mitigation strategies in developing countries. Treating human and non-human actors as separate is contrary to ANT’s principle of symmetry (Ernstson 2008). In context Latour (2005) gives equal status to these two actors, considered interconnected agencies in order to achieve certain goals. For example, when considering the effective utilisation of natural resources (e.g. *Z. mauritiana*) by women in a semi-arid region, one must consider the two actors (humans and the natural resources) without separating them. Since its conception, ANT has continued to extend its efficacy as a tool for studying institutions (Czarniawska & Hernes 2005), tourism and geography (Dwiartama & Rosin 2014), innovation (Ernstson 2008), power (Murdoch 2001) and education (Fenwick & Edwards 2010).

### Power

According to ANT, an actor does not continue to exist merely as an entity but rather as a coalition of heterogeneous essentials that arrange into a network (Latour 2005). Thus, a network is an assemblage of actors and their relations with other actors. Within such a network, it is possible to analyse power relations (Law 1992). The characteristics of a network include the subjective aspect of ‘perspective’ because the ‘actors’ in a network are not merely entities unto themselves but are a more complicated compilation of multiple factors (e.g. lesser actors). *Ziziphus mauritiana*, for example, may be viewed as a functioning hazard mitigation actor that plays a role in a larger network in which it creates associations with and links to other mitigation actors, such as non-governmental actors. The point is that no actor is more important than another (Muhonda et al. 2014; Neisser 2014). Actor–network theory is a versatile tool that can be used to meet the research goals according to the preferred perspective of the researcher (Latour 2005).

Preliminary hazard response efforts by governmental organisations or NGOs tend to focus on food distribution, housing and medical aid, while longer-term assistance and the required social services to sustain assistance are ill-conceived. This frequently occurs as a consequence of hastily conceived responses to previous hazard events, generally in the stature of policies to drive preparedness along by means of mitigation. Various instances overlap and imprecise roles result in conflicts at the expense of the recipients (Quandt et al. 2017). Consequently, within the framework of hazard mitigation is a complexity of interactions – between individuals, groups and communities, as well as government and non-government organisations, each with potentially different concerns and agendas.

### Scale

‘Scale’ is a geographical term used to determine measure and calculate the distance between two points on land (or on a map) in a specific metric. Scale normally represents a distance on a map in relation to the real Earth. Scale is an idea deeply rooted in most geographic thinking as a simple term that refers to proximity or a distance (Blok 2010; Richardson et al. 2017). Actor–network theory refers to scale as networks or associations, which can be either real space or social space (Czarniawska & Hernes 2005). Micro or macro scale: the notion of ‘network’ allows us to dissolve the smaller–larger distinction that has plagued social theory from its inception. Individuals, groups, nations or regions are replaced by connections (Law 1992). It is, therefore, important for researchers to explore how networks change over time.

Hazard mitigation and resilience approaches in most countries are often relegated to the realm of politics and policymakers (Manyanye 2015). Hazard mitigation options have been put forward in policy or by political leaders; hence its development has been surrounded by policy studies (Mileti 1999; Neisser 2014). This seemingly ‘backward’ planning and implementation approach has been referred to as a ‘top–down’ approach, rather than the needed ANT notion of scale. The typical approach has been for actors to focus on hazard mitigation, planned around areas noted on maps to be hazard prone. However, such typical approaches are insufficient as they do not include preparation for hazards through a web of complex connections with different actors, which includes educating the local populace and other actors about potential hazards and effective mitigation techniques.

Actor–network theory is ideally different from the common failed social theory, which calls for a top–down or bottom–up approach. In contrast, it does not attach an assumed relation, be it bottom–up or top–down. The absence of a prior scale makes it easy to follow the changes in the scales. Actors are the determinants of the number, the type and topography of connections. Actor–network theory is essentially interactive, engaging a sense of possession over space, which is not generalisation but actual realities moulded by the deliberate actions of specific actors. Actor–network theory ownership enables a deeper investigation of how individuals express their intervention in space with particular regard to hazard mitigation.

### Network

According to Latour (2005), an ‘actor–network’ is not an ‘object’ but rather ‘collective translations’ and should be viewed as a living, changing entity or a group of interconnected entities. Actors are individual entities; actor–networks are groups of actors: networks of heterogeneous entities linked with one another through different relationships, whose resistance has been overcome (Law 2007). In a network there is a mutual sense of belonging between actors and networks, as networks constitute actors. Actors cannot perform without networks and the reverse is also true. Policies and NGOs cannot provide food aid without networks to fulfil their mission. In the case of *Z. mauritiana*, hazard mitigation strategies are conceptual spaces underpinned by actor-networking in which non-human actors, such as the government and NGOs, are part of the existing network and help in translation, which needs to be followed. Callon (2005) states that it is difficult to recognise the range of effects or impacts of non-human actants as they appear in a number of different ways, for example, natural resources like *Z. mauritiana*, intangible government policies and human-originated food aid packages.

According to Neisser (2014), ANT presents a basic analytical tool, primarily because it has clear ways of exploring the ‘process of translation’ presented by Callon (1986) as follows: firstly, *problematisation* refers to the identification of the problem and possible solutions based on observable facts; secondly, *interessement* implies the support that actants will have towards the identified problem; thirdly, *enrolment* refers to activities that actants perform to solve the identified problems; and fourthly, *mobilisation*, the last moment of translation, refers to the stabilisation in wider or new networks, in which charts, graphs and maps represent the associations and networks of actors as reality (Ernstson 2008). Some social scientists refer to the stability of networks as the ‘black box’. This means when interactions are established, they are durable and remain unquestionable. However, it ought to be mentioned that to have a stable box during the process of translation moments, resistance, negotiation and realignment need to be considered (Dwiartama & Rosin 2014). It is in the interest of this research to understand the four moments of translation and how they relate to the field of resilience and hazard mitigation under study. A network is never bigger than another; it is simply longer or more intensely connected.

### Actants

Actor–network theory uses the term ‘actant’ or ‘actor’ in reference to non-human or human life, which are all treated equally when assigned to some social function (Dwiartama & Rosin 2014; Latour 2005). The primary interest of ANT in practical application is considering the associations or links that may be recognised among various actants for specific purposes, such as policy formulation or research, and the identities, roles and interactions of actants (Law 2007).

Actors come in different forms, though they should be treated equally by the focal actor, who might persuade other actors. Among them are the following (Latour 2005): (1) actors who are not identified by the objectives of the network but are enrolled once in agreement in terms of goals explained by the controlling actor; (2) actors who might be resistant to the roles they are supposed to play; (3) actors who are disruptive and thus act against the interests of the network; and (4) actors who exercise control on behalf of the controlling actor. It must be noted that actors are not actors of a specific network if they do not have an influence on the process of control of a particular network (Latour 2005; Law 1992).

Actor–network theory rejects the notion of human agency (or human intention) as the prime influence on hazard mitigation and recognises all relevant factors (e.g. flora and fauna, water, geologic factors and climate). Latour (2005) notes that the term ‘actors’ in the ANT context is different from the common usage of the term, in which actors are defined as and understood to be single entities. Actor–network theory creates value to any actor because power is bestowed on it. In conclusion, ANT gives insights gained, which will be of use in the understanding of current dynamic social and ecological complexities in alternative hazard mitigation strategies in developing countries.

### Criticism of actor–network theory

Critics question the use of human and non-human actors on an equal footing based on social issues associated with daily routines (Callon & Latour 1992). Actor–network theory does not recognise social structures in a traditional sense but rather places emphasis on the association between entities, which are not recognisable as social in the ordinary manner. There are notable differences in the theory of social network structures, which differ from ANT human components that interact with non-human components to determine power relations among actors (Whittle & Spicer 2008).

Actor–network theory is challenged in its assignment of non-human actors as important facilitators in different processes (Whittle & Spicer 2008). However, advancements in technology have supported proponents of ANT in some ways, because technology plays an important role in many daily activities and shapes them, as supported by Whittle and Spicer (2008).

The question of power has also been probed within a network; however, Latour (2005) believes that power determines the strengths of individuals that might not connect to the ANT. The theory has also been criticised for being ‘everything’, because one cannot tell whether it is a theory or an approach (Whittle & Spicer 2008). The critique is based on the idea that it is too descriptive and fails to deliver concrete suggestions. It has been suggested that ANT should be used with other approaches (Law 2007). Latour, one of ANT’s proponents, stated that it is not and was never intended to be a ‘theory’ but rather a tool with which people and ideas can be connected or assembled. Despite all the criticism, ANT is recognised as a powerful tool and has been used by many researchers (e.g. Law 2007).

### *Ziziphus mauritiana* (or *musawu*)

**FIGURE 3:**
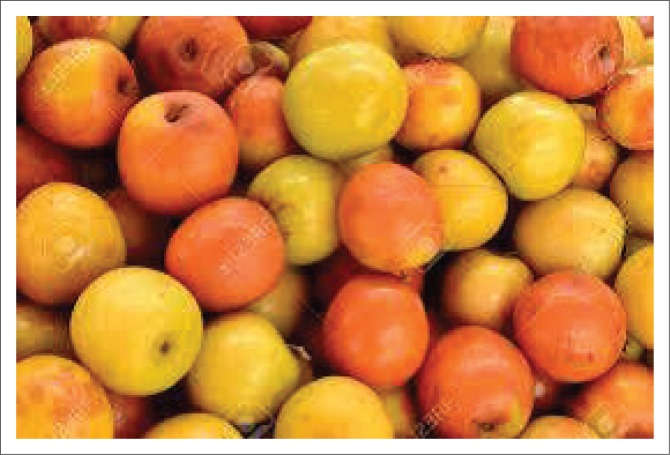
Fresh *masawu* ready for consumption.

*Ziziphus mauritiana* produces edible fruits that are green-yellow when ripened and eventually turn brownish when dried (see Figure 3).

*Ziziphus mauritiana* is part of the savannah forest patterns that forge mutually valuable associations, creating a rural ecosystem that is more than the parts of its sum (Bodin, Crona & Ernstson 2006). In Muzarabani, *Z. mauritiana*, an indigenous fruit tree that provides important sustenance during periods of hazard, exemplifies a non-human actor in ANT (Neisser 2014). *Ziziphus mauritiana* provides fruits that can be used as a source of food, fuel, medicines and possibly other as yet unknown products. Human activities, however, threaten the sustainability of this rich natural resource (Saka et al. 2007). In the same context *Z. mauritiana* offers ecological, economic and social roles that include the provision of employment, acting as a food safety net for communities (Thondhlana & Shackleton 2015) in Muzarabani, even in other times of hardship, for example, high unemployment and general impoverishment in the community. *Ziziphus mauritiana* is eaten fresh and dried. It can be used as a fruit drink, non-alcoholic and alcoholic beverages. It is a cheap source of different vitamins, especially C, is rich in sugar and provides a reliable animal dietary source for domestic local livestock (Saka et al. 2007). It presents a food safety net when the region experiences weather catastrophes like floods (Shackleton & Gumbo 2010). Despite its importance, *Z. mauritiana* has received little or no attention, even at policy level, when dealing with issues in fragile environments like Dande. *Ziziphus mauritiana*, locally known as *musawu*, provides an opportunity for an actor–network analysis, which helps to explain the complex mutual association of non-human and human actors in the hazard mitigation discourse on Muzarabani (Neisser 2014).

The rural Muzarabani community relies heavily on *Z. mauritiana*, which arguably has its origins in India and is currently naturalised in many tropical regions (Mukhtar et al. 2004). Countries where *Z. mauritiana* is grown naturally and domestically include Russia, the United States, China and countries in the Middle East. *Ziziphus mauritiana* is known in India as *jujube* or *desert apple*. It is a tropical fruit, normally cultivated in marginal lands. Small and large plantations have been observed in Afghanistan (Mukhtar et al. 2004). The absence of these in Zimbabwe and other sub-Saharan African countries remains a puzzle. Muzarabani, like most rural areas in semi-arid regions south of the Sahara, is characterised by complex and intricate social, ecological and political webs between and among different actors (Tantoh & Simatele 2017). This makes addressing resilience and hazard mitigation strategies a daunting task. Primary reasons for mitigation strategy failures are multifaceted and include political interference, non-existent institutions, weak policies on hazard mitigation, lack of local support and insufficient manpower, monitoring and evaluation (Muhonda et al. 2014). Concurrently, demonstrated success in Muzarabani could provide viable mitigation planning information to other regions and countries. The analyses of the value attached to *Z. mauritiana* by different actors could be explained by the commodity value chain approach.

### Commodity chains

It is imperative to follow the identified eco-resource through literature to deduce meaning that would benefit the poor in semi-arid regions. The commodity chain approach has been used extensively in social science scholarly work (Chibarabada, Mabhaudhi & Modi 2017). The concept ‘commodity chain’ in this paper refers to the analytical overview that defines specific arrangements among actors that confirm the flow of *Z. mauritiana* from the input resources used to the end user or consumer. It is well documented that placing commodities at the hub of the narrative builds the approach’s muscle, not only in tracing the material flow of commodities but in following the social relations flow. The commodity chain is an important tool used to study and identify actors, spaces and relations. The chain also identifies and explains sites of production, barter and use. In addition, the commodity chain goes beyond the nodes and institutions of specified markets; commodity chain analysis recognises points of regulation and power among various actors (Chibarabada et al. 2017; Saguin 2014).

Concepts that are related but theoretically different are *filière*, global commodity chain (GCC) and global production network (GPN). The identified concepts all give explanations of the relationships, business and conduct involved when commodities move. Intellectual bias, themes and methods are the origins of concepts that constitute the value chain. Very few scholars discuss the *filière*, which is entrenched in the societal analysis of the home production-use dynamics of agro-ecological commodities (Saguin 2014). *Filière* is argued to be the most traditional concept to explain value chain. Recent scholars do not mention or use the concept *filière*; instead they use the terms GCC and GPN (Chibarabada et al. 2017). GPN, unlike the traditional concept *filière*, are more concerned with global chains or associations that are entangled in production, generally in the framework of export or manufacturing industries (Saguin 2014). Though regarded as traditional, it is useful and compatible with current concepts. *Filière* can be supported with commitment from current concepts and methodologies, namely GCC and GPN.

Literature has it that commodity chain concepts in discourse within a given approach offer likely benefits. According to Gereffi and Fernandez-Stark (2011), the GCC emphasises input–output framing by the actors involved. Global commodity chain complements trade among agro-ecological actors. Focus will be on determining material flows in different spaces (Gereffi et al. 2005; Gereffi 2011). Global commodity chain, however, goes further than mere accounting by collectively embedding the flows of material spaces (Grivins 2016) and focusing on power relations within the chain, a primary concern of hazard mitigation priorities. In context, the change in these flows and chain should be analysed within the broader context of the fruit (*Z. mauritiana*) production. The study considers the strengths of the GPN-based methodology in formative distributional concerns within the chain, predominantly those enshrined in inter-actor linkages (Grivins 2016). The idea of linear chain thinking has been criticised by the GPN approach for its limitations. The complexities of internetwork linkages within the value chains call for a different lens as proposed by the GPN (Chibarabada et al. 2017).

The social ecological orientations of economic activities have influenced the GCC and GPN frameworks (Grivins 2016). While the actors’ explanation in this article mentions equity, responsibilities and other social associations fit within this practice. The aim of this review is to give a background into the enquiry of actors, access, control and interlinkages. The ANT framework (Murdoch, Marsden & Banks 2000) is of use here as a supplement, even if it is criticised for not addressing the structural sources of power, as put forward by Grivins (2016) in a commodity chain approach. Studies grounded on ANT have the potential to extend our understanding of actors and who or what is entangled in the creation of commodity networks (Fink & Weyer 2014). However, these scholarships lean towards restricting their analyses to particular networks, engaged in how the action of actors materialise because of their interactive roles within these linkages and how these protagonists establish and alter the linkage under study. These scholarships do not reflect how contestants in commodity linkages are involved in other classes of social interactions, nor do they mirror the conducts in which their involvement is formed by wider cultural accounts and associations of power (Manyanye 2015).Therefore, these studies also fail to capture the various, positioned sets of eco-cultural associations through which commodity chains take form for the benefit of the poor communities in natural hazard-prone areas.

## Research gap and justification of the study

Hazard mitigation strategies are meant to reduce loss of life and property; regrettably, the real world is burdened with recurrent hazards (Kenney et al. 2015), repeatedly worrying the same community in a menacing and harsh way. However, there is limited empirical research on hazard mitigation approaches that place actor–network theory into perspective (Neisser 2014). Actor–network theory has been used successfully in other disciplines like tourism, information and technology (Murdoch 2001); however, it has scarcely been used to enhance hazard mitigation strategies in Zimbabwe, sub-Saharan Africa and the world. The structural and non-structural hazard mitigation initiatives in most cases have failed to yield the intended results.

Research on hazards has been conducted (Ernstson 2008), but very little seems to provide comprehensive enhancement of hazard mitigation approaches underpinned by complex socio-ecological networks. One of the most compelling reasons for choosing to focus on *Z. mauritiana* is that in this particular Muzarabani district, it is such an important part of the daily lives of its inhabitants. It is ‘intimately linked’ to this non-human actor (Kadzere & Jackson 1998). It is widely suggested that there is a need to incorporate different actors, both human and non-human, in hazard mitigation options. It is imperative to comprehend their complexities and the actor networks that can stabilise hazard mitigation and resilience options.
